# Immunogenicity and protective efficacy of a multi-antigenic adenovirus-based vaccine candidate against *Mycobacterium tuberculosis*

**DOI:** 10.3389/fmicb.2025.1492268

**Published:** 2025-01-24

**Authors:** Jin-Seung Yun, Eunkyung Shin, Young-Ran Lee, Jung-Ah Lee, Hyeokjin Lee, Jong-Seok Kim, Sung Jae Shin, Sang-Jun Ha, Sang-Won Lee, Dokeun Kim, Jung-Sik Yoo, Hye-Sook Jeong

**Affiliations:** ^1^Korea National Institute of Health, Korea Disease Control and Prevention Agency, Cheongju, Republic of Korea; ^2^Department of Biochemistry, College of Life Science and Biotechnology, Yonsei University, Seoul, Republic of Korea; ^3^Bio-Convergence R&D Division, Korea Institute of Ceramic Engineering and Technology, Cheongju, Chungbuk, Republic of Korea; ^4^Korea Disease Control and Prevention Agency, Cheongju, Republic of Korea; ^5^Department of Cell Biology, College of Medicine, Myunggok Medical Research Institute, Konyang University, Daejeon, Republic of Korea; ^6^Department of Microbiology, Graduate School of Medical Science, Brain Korea 21 Project, Yonsei University College of Medicine, Seoul, Republic of Korea

**Keywords:** pulmonary tuberculosis, BCG vaccine, adenovirus vector, multi-antigenic vaccine, immunization, mouse model, immune response

## Abstract

**Introduction:**

The inadequate efficacy of the Bacillus Calmette–Guérin (BCG) vaccine against adult pulmonary tuberculosis (TB) necessitates the development of new and effective vaccines. Human adenovirus serotype 5 (Ad5), which induces T-cell response, is a widely used viral vector. In this study, we aimed to evaluate the efficacy of a multi-antigenic recombinant Ad5 vectored vaccine and determine the optimal immunization route for enhanced immune response against *Mycobacterium tuberculosis*.

**Methods:**

We constructed a multi-antigenic recombinant Ad5 vectored vaccine expressing four antigens (Ag85B-ESAT6-MPT64-Rv2660c) of *M. tuberculosis* (rAd-TB4), immunized with rAd-TB4 (5 × 10^7^ infectious virus units/mouse) twice at an interval of 4 weeks starting at 10 weeks after BCG priming, and evaluated its boosting efficacy in a BCG-primed mouse model, and determined the optimal immunization route.

**Results:**

Compared with the BCG-only (2 × 10^5^ colony forming units/mouse), subcutaneous injection of rAd-TB4 (1 × 10^7^ infectious virus units/mL; two doses) elicited a T-cell response and cytokine production in lung lymphocytes and splenocytes. rAd-TB4 immunization significantly reduced bacterial loads and inflamed lung areas compared to BCG immunization (*p* < 0.01) and protected against the H37Rv challenge performed at 17 weeks of BCG priming. RNA sequencing of the whole blood of rAd-TB4-vaccinated mice collected pre- and, 1 and 4 weeks post-infection, identified differentially expressed genes associated with immune and inflammatory responses, especially those in the Wnt signaling pathway.

**Conclusion:**

Our results indicate that rAd-TB4 immunization enhances the immune response to the vaccine boosting antigens in BCG-primed mice, making it a potential adult pulmonary TB vaccine candidate.

## 1 Introduction

Tuberculosis (TB), caused by *Mycobacterium tuberculosis*, a communicable disease, is among the top ten causes of death worldwide (Bagcchi, 2023). Annually, an estimated 10.0 million people are infected with *M. tuberculosis* and develop TB during their lifetime (Bagcchi, 2023). According to the World Health Organization TB report in 2023, approximately 90% of people who develop TB are adults (Bagcchi, 2023). Bacille Calmette–Guérin (BCG), the attenuated form of *Mycobacterium bovis*, is the only licensed vaccine against TB that prevents meningitis and miliary disease in children; however, its efficacy diminishes 10–15 years after vaccination during adolescence and in adults (Trunz et al., 2006; Abubakar et al., 2013; Hatherill and Cobelens, 2022) rendering the adults unprotected against pulmonary TB. Therefore, the development of a more effective vaccine to improve the pulmonary immune responses to *M. tuberculosis* in adults has remains of substantial clinical and research interest.

Among the promising vaccine platforms, adenovirus vectors effectively activate T-cells and are widely employed as vaccine carriers for various diseases and pathogens, such as human immunodeficiency virus type-1 (Barouch, 2010) and *Plasmodium falciparum* (Sharma et al., 2010; McGuire et al., 2017), respectively. A chimpanzee adenoviral-vectored vaccine (ChAdOx185A), heterologously immunized with modified vaccinia Ankara 85A (MVA85A), is considerably effective when administered intranasally. Moreover, a phase IIa study on the efficacy of this vaccination strategy via intramuscular (IM) administration in adults and adolescents is ongoing (Stylianou et al., 2015; Wilkie et al., 2020). Furthermore, recombinant human type 5 adenovirus (HAd5)-vectored vaccines expressing the dominant antigens of *M. tuberculosis* (AdHu5Ag85A) have been shown to be effective in both BCG-naïve and BCG-immunized healthy volunteers in Canada (Smaill et al., 2013).

Despite their high efficacy, adenovirus-vectored vaccines face issues in clinical trials because the immune response to HAd5-based vaccines can be blunted by the pre-existing immunity acquired from natural exposure (Fausther-Bovendo and Kobinger, 2014; Hassan et al., 2017). However, this pre-existing immunity considerably decreases with periodic or heterologous immunization (Yoshida et al., 2018; Vierboom et al., 2020). In addition, HAd5-based vaccines elicit enormous T-cell responses in humans despite pre-existing immunity (McCoy et al., 2007).

During mycobacterial infection, type-1 T-helper (Th1) cell immunity plays a crucial role in host protection (Woodworth et al., 2017; Soto et al., 2018). Th1 cells produce interferon-γ (IFN-γ), tumor necrosis factor-α (TNF-α), and interleukin-2 (IL-2), which are the major pro-inflammatory cytokines implicated in pathogen eradication via opsonization and phagocytosis (Darrah et al., 2007). However, so far no T cell assay correlates with protection in humans (Dockrell and Smith, 2017) and a protective role has also been attributed to antibodies (Dieli and Ivanyi, 2022). Several TB vaccines target Th1 cell immunity using mycobacterial immune-dominant antigens (Lewinsohn et al., 2017; Rodo et al., 2019; Kaveh et al., 2020). These data justified to assume, that a T-cell immune response-inducible vaccine could efficiently improve the pulmonary immune responses to *M. tuberculosis* in adults.

Fourteen vaccine candidates currently under clinical trials express immunogenic antigens against *M. tuberculosis* on various platforms (Cobelens et al., 2022). Among these, the antigen 85 (Ag85) complex (Ag85A, Ag85B, and Ag85C), early secreted antigenic target, 6 kDa (ESAT-6), and *M. tuberculosis* complex protein 64 (MPT64) secreted during the early phase (Brandt et al., 2000; Huygen, 2014; Babaki et al., 2017; Cao et al., 2021) and Rv2660c associated with latent phase (Yihao et al., 2015) play important roles in the survival of *M. tuberculosis* within macrophages and are abundant during their respective infection phases. In addition, they activate Th1 cell immunity to produce IFN-γ, TNF-α, and IL-2 (Aagaard et al., 2011; Leung-Theung-Long et al., 2015). Previous studies have also shown that multi-antigenic vaccines, such as MVATG18598 and AERAS-402, aim to elicit T-cell responses with antigens expressed in various phases (Abel et al., 2010; Tameris et al., 2015; Lauer et al., 2017; Leung-Theung-Long et al., 2018). Therefore, we hypothesized that a T-cell immune response-inducible vaccine incorporating several immunogenic antigens of *M. tuberculosis* could establish an enhanced efficacy against pulmonary TB in adults.

To test this hypothesis, in this study, we developed a multi-antigenic recombinant Ad5 vaccine candidate—rAd-TB4—by incorporating the key immunogenic antigens—Ag85B-ESAT6-Mpt64-Rv2660c—using the HAd5 viral vector platform and evaluated its protective efficacy in a BCG-primed mouse model. Furthermore, we evaluated the potency of rAd-TB4 to protect against the H37Rv challenge and analyzed the changes in the associated transcriptome in a BCG-primed–rAd-TB4-boosted and H37Rv-infected mouse model. This study provides useful insights into the boosting effect of a multi-antigenic vaccine candidate against adult pulmonary TB.

### 2 Materials and methods

### 2.1 Animals

Four to five-week-old female C57BL/6 mice were purchased from DooYeol Biotech (Seoul, Korea). Mice were kept under standard environmental conditions and with ad libitum access to commercial food and tap water. All mouse studies were performed in accordance with the grant by the Laboratory Animal Welfare and Ethics Committee, Korea Disease Control and Prevention Agency (KDCA), in compliance with Institutional Animal Care and Use Committee guidelines for the care and use of animals (permit number: KCDC-124-19-2A). All processes complied with ARRIVE guidelines and American Veterinary Medical Association Guidelines for the Euthanasia of Animals.

### 2.2 Construction of rAd-TB4

The multi-antigenic recombinant antigen was constructed following the procedure described in a previous study (Zhou et al., 2018). Briefly, whole sequences of Ag85B, ESAT-6, a partial sequence of MPT64 (190–198 residues), and Rv2660c antigens of *M. tuberculosis* were obtained from Mycobrowser^1^ and cloned into the pAd/CMV/V5-DEST vector (Invitrogen, Waltham, MA, United States) in order and linked using an Asp-Val-Ala and Gly-Ser-Gly linker to generate the candidate rAd-TB4 (Supplementary Figure S1A).

Subsequently, to confirm the construction of the adenovirus vector vaccine rAd-TB4, we analyzed its protein expression using western blotting. The culture supernatant and cell lysates obtained after infection were analyzed using a polyclonal-antibody specific to Ag85B, ESAT-6, MPT64 (Abcam, Cambridge, United Kingdom), and purified protein derivative (PPD; Mybiosource, San Diego, CA, United States). Briefly, HEK293A cells (Invitrogen) were grown in Dulbecco’s modified Eagle’s medium (DMEM, Gibco, Grand Island, NY, United States) containing 10% fetal bovine serum (FBS, Gibco) and 1% penicillin/streptavidin (P/S, Gibco) at 37°C for 24 h (Davis et al., 2001). Freshly grown HEK293A cells were then transfected with rAd-TB4 and cultured under DMEM containing 2% FBS at 37°C for 24 h. When approximately 80% of the cells displayed cytopathic effects, the infected cells and supernatants were collected to isolate the virus. The virus was purified and concentrated using a Virabind Adenovirus Purification Kit (Cell Biolabs, Inc., San Diego, CA, United States) following the manufacturer’s instruction, stored at -80°C, and titrated using the Adeno-X Rapid Titer Kit (Takara Bio, Japan). HEK293A cells were used as a negative control, and the Ag85B protein was used as a positive control for the Ag85B antibody.

### 2.3 Preparation of *M. bovis* BCG and *M. tuberculosis* cultures

*M. tuberculosis* Strains H37Rv and HN878, were obtained through BEI Resources, NIAID, NIH (NR-123 and NR-13647, respectively); BCG Pasteur 1173P2 was provided by the KDCA and grown in a medium containing Middlebrook’s 7H9 broth (Difco Laboratories, Detroit, MI, United States) supplemented with 10% Oleic Albumin Dextrose Catalase growth enrichment (Becton Dickinson, Sparks, MD, United States) and 0.2% glycerol at 37°C on a shaker at 200 rpm under aerobic conditions for 14–20 days (Cole et al., 1998). To prepare single-cell suspensions, mycobacterial cells were harvested via centrifugation at 10,000 × *g* for 20 min and washed thrice with PBS (pH 7.2). The pellets were passed through 40-, 20-, 10-, and finally, 8-μm filters (Millipore Corp., Burlington, MA, United States). The final stock was stored in small aliquots at −80°C until further use. Colony-forming units (CFUs) per milliliter of stock were measured using a counting assay on 7H10 agar plates.

### 2.4 Animal immunization and aerosol infection

Female mice (5–6 weeks old; n = 5) were vaccinated with BCG Pasteur 1173P2 (2 × 105 CFUs/mouse) subcutaneously (week 0), followed by immunization with rAd-TB4 (5 × 10^7^ infectious virus units/mouse) twice at 10 and 14 weeks after BCG priming via subcutaneous (SC) or IM injections. One week after the final immunization (at 15 weeks), the mice (*n* = 5) were euthanized via CO_2_ inhalation to analyze immunogenicity. The measurement of immune response in the lung lymphocytes and splenocytes of rAd-TB4-administered mice was assessed by measuring *ex vivo* responses using the ELISpot assay, intracellular cytokine staining, and immunoglobulin G (IgG) titer measurements. Briefly, single lung lymphocyte and splenocyte cells were stimulated with different antigens—rAd-TB4 (Ag85B, Rv2660c, and ESAT-6) or PPD—and IFN-γ production per 1 × 10^6^ cells was determined using the ELISpot assay. For single-cell preparation, the spleens and lungs were aseptically collected and pooled for each group. Spleens were homogenized for immune assays using a gentleMACS μTissue Dissociator (Miltenyl Biotec, Germany), washed in RPMI-1640 medium (GenDEPOT, Baker, TX, United States) supplemented with 10% FBS and 1% P/S, rinsed in ammonium–chloride–potassium buffer to remove erythrocytes, and passed through a 40-μm cell strainer to generate single splenocytes. Lung tissues were incubated with DNase I (Roche, Swiss) and collagenase D (Roche) in a plain medium at 37°C for 1 h to isolate lymphocytes from mouse lungs. Lymphocytes were separated from 5 to 20 mL of lung cell suspension on a Lymphoprep gradient (STEMCELL Technologies, Canada) using density centrifugation, passed through a 40-μm cell strainer and resuspended in DMEM containing 10% FBS and 1% P/S.

Three weeks after the final immunization (at 17 weeks), another batch of mice was challenged with the *M. tuberculosis* H37Rv strain (experiment 1: *n* = 5; experiment 2: *n* = 3) or HN878 (*n* = 6) strain using a Glas-Col aerosol generator (Glas-Col LLC., Terre Hautre, IN, United States). The infection conditions were calibrated to expose each mouse to approximately 200 CFU following the procedures described in a previous study (Kwon et al., 2019). To estimate antigen specific IgG and subtypes, whole blood from the mice n the experiment 1 batch was collected eight weeks post-challenge and centrifuged to obtain serum.

### 2.5 Multifunctional T-cell assay

For multifunctional T cell analysis, single cells (2 × 10^5^) were stimulated with PPD (NIBSC, Blanche Lane, UK), ESAT-6, Ag85B, or Rv2660c peptide (100 ng/mL; JPT Peptide Technologies GmbH, Berlin, Germany) for 5 h at 37 °C in the presence of GolgiPlug and GolgiStop (BD Biosciences, East Rutherford, NJ, United States). Stimulated cells were washed with PBS containing 3% bovine serum albumin (BSA; Sigma Aldrich, Saint Louis, MO, United States) and stained with FITC-conjugated anti-MHC II, V450-conjugated anti-CD4, and PerCP-Cy5.5-conjugated anti-CD8 antibody for 30 min at 4°C. The cells were then permeabilized with the Fixation/Permeabilization Solution Kit (BD Biosciences) for 30 min at 4°C, intracellularly stained with APC-conjugated anti-IFN-γ, PE-Cy7-conjugated anti-TNF-α, and PE-conjugated anti-IL-2 antibody for 30 min at 4°C, fixed with 4% paraformaldehyde, and resuspended in PBS containing 3% BSA. Analyses were performed using FACSVerse (BD Biosciences) and FlowJo software v9 (BD Biosciences) to gate triple-, double-, or single-positive T-cell populations (Supplementary Figure S2). Data are expressed as pie charts with the percentage of positively gated T-cells among CD4- or CD8-positive (CD4^+^ or CD8^+^) populations. For the detection of memory T cells and activated phenotypes, lymphocytes were stained with BV605-conjugated anti-CD3 (BD Biosciences), PerCP-Cy5.5-conjugated anti-CD4 (BD Biosciences), PE-conjugated anti-CD44 (eBioscience, San Diego, CA, United States), FITC-conjugated anti-CD62L (eBioscience), and APC-conjugated anti-CD127 antibody (eBioscience) for 30 min at 4°C, fixed, and resuspended in PBS containing 3% BSA. Analyses were performed using identical equipment and software.

### 2.6 ELISpot assay for IFN-γ secretion

The ELISpot assay was performed using an IFN-γ secretion ELISpot kit. Briefly, a single-cell suspension (5 × 10^5^ cells) was stimulated with PPD, Ag85B, Rv2660c, and ESAT-6 peptide (100 ng/mL) for 36 h at 37°C in anti-IFN-γ antibody-coated filter plates. Subsequently, biotinylated anti-IFN-γ antibody, streptavidin-horseradish peroxidase (HRP) conjugate, and 3-amino-9-ethylcarbazole were added as substrates to develop secreted cell spots, which were quantified using an Immunospot S6 analyzer (Cellular Immunospot Limited, Shaker Heights, OH, United States). The results are presented as mean values of triplicate wells for each group. All substrates and the ELISpot kits were purchased from BD Biosciences.

### 2.7 Quantification of cytokines

A single-cell suspension (5 × 10^5^ cells) was stimulated with Ag85B peptide for 36 h at 37°C, and the supernatant was collected to measure cytokine expression. Supernatants were diluted in a 1:2 ratio with complete RPMI medium and assayed in triplicate wells of each group using the Bio-Plex Pro Mouse Cytokine 10-plex, which was customized to include IFN-γ, TNF-α, IL-2, Th17-related cytokine (IL-17A), GM-CSF, IL-6, IL-12p40, IL-12p70, and IL-10 (Bio-Rad Laboratories, Hercules, CA, United States), following the manufacturer’s instructions. The results were acquired using a Bio-Plex MAGPIX reader (Bio-Rad Laboratories), and the mean values were used to plot graphs.

### 2.8 Measurement of serum IgG titer

We coated 96-well flat-bottom Immuno Plates (Thermo Fisher Scientific, Waltham MA, United States) with 100 ng/mL PPD, Ag85B, Rv2660c, or ESAT-6 for 18 h at 4°C. After washing with PBS containing 10% tween-20, each well was blocked with PBS containing 3% BSA for 2 h at 37°C. Serum samples were diluted at 1:200 with PBS containing 3% BSA and incubated for 2 h at 37 °C. After washing, a 1:2,000 dilution of goat anti-mouse IgG-HRP (Thermo Fisher Scientific) was added and incubated for 1 h at 37 °C. In the case of challenged mice serum, goat anti-mouse IgG1-HRP (Thermo Fisher Scientific) and goat anti-mouse IgG2c-HRP (Abcam) were additionally used. The substrate, tetramethylbenzidine (TMB; Thermo Fisher Scientific), was added to each well, and the plate was incubated at 37 °C for 15–30 min. Thereafter, a stop solution for TMB was added, and the plates were read using a spectrophotometer (Spectramax i3x, Molecular Devices, San Joes, CA, United States) at 450 nm.

### 2.9 CFU and histopathology

To estimate the CFUs of *M. tuberculosis* in the lungs of infected mice, the lungs were homogenized in 3 mL of PBS at 12 weeks post-challenge. Subsequently, 10-fold dilutions of the tissue homogenates were plated on 7H11 Middlebrook Agar (Difco Laboratories). The plates were incubated at 37°C for 3–4 weeks, and the number of colonies was determined to assess the total CFUs in the lungs. For histopathological analysis, the lungs of infected mice were fixed in formalin (Sigma Aldrich) and embedded in paraffin. Paraffin blocks were cut and stained with hematoxylin and eosin (H&E). A Motic Easy Scan scanner was used to scan the histological sections, and the images were analyzed to quantify the granulomatous inflammation. The percentage of granulomatous tissue in the whole lung was calculated using the ImageJ software version 1.53j. The experiment was conducted twice using the identical mouse immunization and challenge schedule, CFU estimation, and granulomatous inflammation quantification as described above.

### 2.10 RNA isolation and data analysis

Total RNA was isolated using TRIzol reagent (Invitrogen) and RNA quality was evaluated using an Agilent 2100 bioanalyzer using an RNA 6000 Nano Chip (Agilent Technologies, Amstelveen, The Netherlands). RNA was quantified using an ND-2000 Spectrophotometer (Thermo Fisher Scientific), according to the manufacturer’s instructions. An RNA library was constructed using a QuantSeq 3′ mRNA-Seq Library Prep Kit (Lexogen, Inc., Austria) according to the manufacturer’s protocol. High-throughput single-end 75-bp sequencing was performed using NextSeq 500 (Illumina, Inc., San Diego, CA, United States). QuantSeq 3′ mRNA-Seq reads were aligned using Bowtie2 (Langmead and Salzberg, 2012). The alignment file was used to assemble transcripts, estimate their abundance, and detect differentially expressed genes (DEGs) based on counts from unique and multiple alignments using coverage in BEDTools (Quinlan and Hall, 2010). The read count data were processed based on the quantile normalization method using EdgeR within R with the help of Bioconductor (Gentleman et al., 2004). Statistically significant DEGs were arranged into hierarchical clusters using correlation distance and presented in a heatmap. All correlation analyses employed Pearson correlation coefficients, and the Benjamini-Hochberg method was applied for multivariate analysis adjustments, setting the false discovery rate (FDR) threshold at < 0.25. Principal component analysis (PCA) was employed to demonstrate the similarities and differences between samples in the dataset and to detect any outliers. After obtaining a ranked list of DEGs, Gene Ontology (GO) enrichment analysis was performed to categorize and annotate the genes into groups such as a biological process using the Database for Annotation, Visualization, and Integrated Discovery (DAVID)^2^ (Ashburner et al., 2000; Lovero et al., 2022; Sherman et al., 2022).

### 2.11 Statistical analyses

All graphical visualization and statistical tests were done using GraphPad Prism v10 (GraphPad Software, La Jolla, CA, United States). The statistical significance between respective antigen restimulation was determined with an unpaired Student’s t-test. One-way analysis of variance using Dunnett’s multiple comparison test was used to evaluate significance differences between more than two vaccine groups. The differences with a *p*-value < 0.05 were considered significant. Data expressed in graphs are presented as the mean ± standard deviation.

## 3 Results

### 3.1 Development of rAd-TB4 vaccine and evaluation of vaccine induced immunogenicity

The candidate rAd-TB4 multi-antigenic recombinant vaccine was developed by inserting Ag85B, ESAT6, MPT64, and Rv2660c antigens into the pAd/CMV/V5-DEST vector (Supplementary Figure S1A). Its protein expression was confirmed using a poly-antibody specific for Ag85B, ESAT-6, MPT64, and PPD in infected HEK293A cells cultured supernatant and lysates (Supplementary Figure S1B).

**FIGURE 1:**
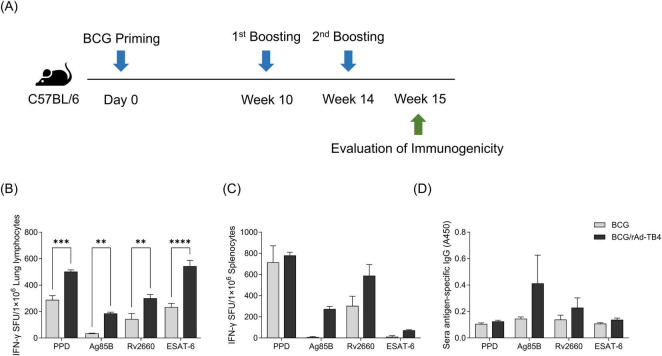
Evaluation of rAd-TB4 immunogenicity as a Bacille Calmette–Guérin (BCG) booster vaccine. **(A)** Schematic of the study design. C57BL/6 mice were subcutaneously vaccinated with BCG (2 × 10^5^ CFU/mouse) on day 0. The mice were immunized with rAd-TB4 (1 × 10^7^ IFU/mouse) twice at 10 and 14 weeks, and then at 15 weeks, five mice in each group were sacrificed to collect their lungs, spleens, and sera. Immunogenicity was evaluated using an enzyme-linked immunosorbent spot (ELISpot) assay and immunoglobulin G (IgG) titer measurement. **(B–D)** Interferon-γ (IFN-γ) secretion in **(B)** lung lymphocytes and **(C)** splenocytes detected using the ELISpot assay following 36 h of incubation of single cells with purified protein derivative (PPD), Ag85B, Rv2660c, and ESAT-6 peptide (100 ng/mL). The BCG group is presented as a gray bar and the rAd-TB4 group was presented as a black bar. **(D)** Antigen-specific total IgG titer in serum samples measured using enzyme-linked immunosorbent assay (ELISA). Data show the mean ± standard deviation from triplicate wells in each group; *p < 0.05, **p < 0.01, ***p < 0.001, ****p < 0.0001 obtained using ordinary two-way ANOVA.

Next, we evaluated the immunogenicity of the rAd-TB4 vaccine candidate by analyzing the T-cell response induced by it. First, C57BL/6 mice were subcutaneously vaccinated with BCG, followed by immunization with two doses of rAd-TB4 vaccine (Figure 1A). Compared with the BCG alone immunization, heterologous immunization with BCG priming and rAd-TB4 boosting highly induced IFN-γ secretion in response to stimulation with all antigens (Ag85B, Rv2660c, and ESAT-6) and PPD in lung lymphocytes (Figure 1B). rAd-TB4-immunization increased IFN-γ secretion in splenocytes upon stimulation with the antigens and PPD compared to BCG immunization, and the difference was significant in cells stimulated with Ag85B and ESAT-6 (Figure 1C). Furthermore, in mice vaccinated with BCG/rAd-TB4, the TB antigen-specific IgG titer was higher than that in those vaccinated with BCG alone in Ag85B- and Rv2660c-specific response, and there was no difference in PPD and ESAT-6 specific IgG titer (Figure 1D).

**FIGURE 2:**
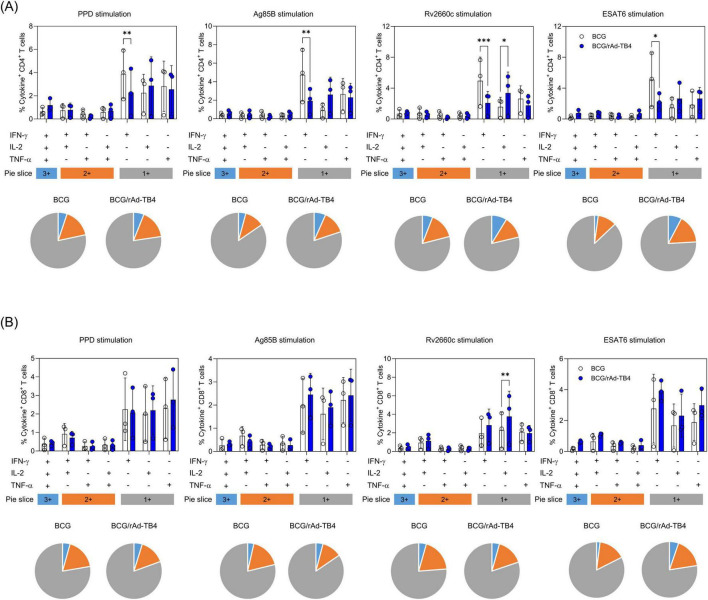
Antigen-specific multifunctional T-cells in the lung lymphocytes of rAd-TB4- and BCG-only immunized mice. One week after the final immunization (see Figure 1A), the mice in BCG-only and BCG-primed-rAd-TB4 immunized groups (*n* = 5 in each) were sacrificed, and their lungs were collected for intracellular cytokine staining assays to evaluate multifunctional T-cells. Lung lymphocytes from each group were stimulated with PPD, Ag85B, Rv2660c, and ESAT-6 peptides (100 ng/mL) for 5 h at 37°C in the presence of GolgiPlug and GolgiStop. Data are shown as the intensity of **(A)** CD4^+^ or **(B)** CD8^+^ T-cells expressing one, two, or three specific cytokines upon (i) PPD stimulation, (ii) Ag85B stimulation, (iii) Rv2660c stimulation, (iv) ESAT-6 stimulation in each group. Data show the mean ± standard deviation from triplicate wells in each group; *p < 0.05, **p < 0.01, ***p < 0.001 obtained using ordinary two-way ANOVA.

To assess CD4^+^ and CD8^+^ T-cell responses to rAd-TB4 immunization, we used ICS to quantify cytokine frequencies. Ag-specific stimulated T-cells were stained with intracellular Th1-type cytokines (IL-2, TNF-α, and IFN-γ) and separated into triple-, double-, or single-positive populations based on a combination of secreted cytokines. Lung lymphocytes in rAd-TB4-immunized mice contained a higher proportion of triple-positive multifunctional CD4^+^ T-cells than those in BCG-immunized mice (Figure 2A). Similarly, the CD8^+^ T-cell population showed numerous triple- and double-positive T-cells in the rAd-TB4-immunized group (Figure 2B). These results suggest that rAd-TB4 promotes CD4^+^ and CD8^+^ T-cell responses and implicates the induction of *M. tuberculosis*-specific IgG production.

### 3.2 Optimal route for rAd-TB4 administration in BCG-primed mice

**FIGURE 3:**
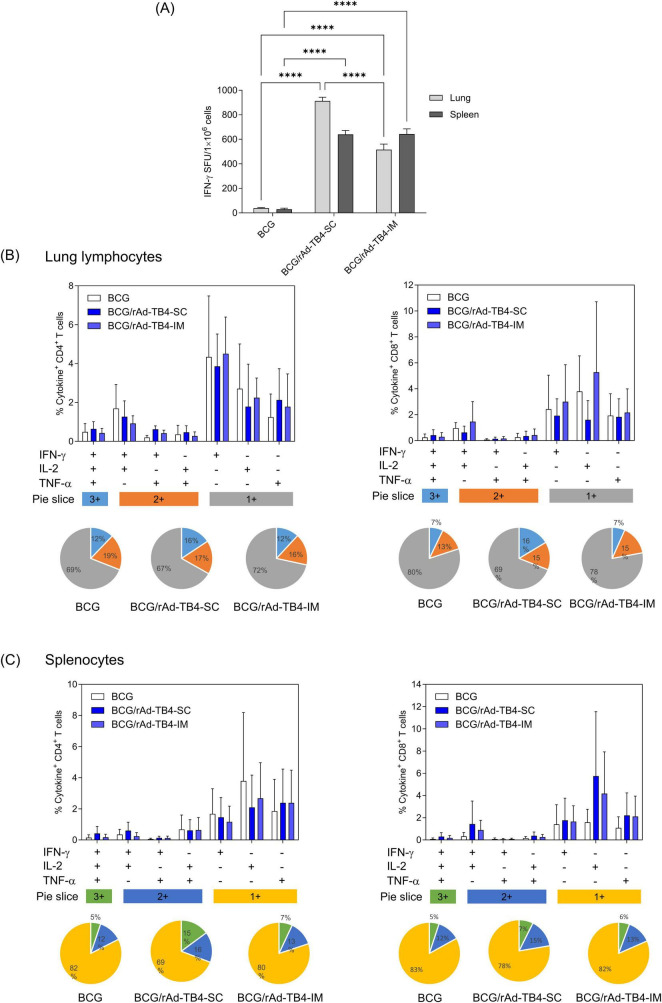
Immunogenicity of rAd-TB4 injected using different routes in BCG-primed mice. The mice were divided into BCG-only and BCG-primed- rAd-TB4 immunized groups. BCG/rAd-TB4 group was further divided into two groups depending on the route of rAd-TB4 administration—subcutaneous (SC) or intramuscular (IM). **(A)** IFN-γ secreted by single cells of mice immunized via the two routes detected using the ELISpot assay following 36 h of incubation with the Ag85B peptide (100 ng/mL). **(B,C)** Cytokines (%) and CD4^+^ or CD8^+^ T-cells in **(B)** lung lymphocytes and **(C)** splenocytes of the respective groups stimulated with Ag85B peptide for 5 h at 37°C in the presence of GolgiPlug and GolgiStop. Data are shown as the intensity of CD4^+^ (left) or CD8^+^ (right) T cells expressing one, two, or three cytokines in each group. Statistical analysis was performed using the unpaired Student’s *t*-test; **p < 0.01, ***p < 0.001, compared with the BCG group. Data show the mean ± standard deviation from triplicate wells in each group; *p < 0.05, **p < 0.01, ***p < 0.001, ****p < 0.0001 obtained using ordinary two-way ANOVA.

Establishing an appropriate regimen is a crucial step in investigating vaccine efficacy. Therefore, we determined the optimal route of administration and dose to establish the maximum benefits of rAd-TB4, which showed high immunogenicity in BCG-primed mice. Mice were vaccinated with rAd-TB4 via SC (BCG/rAd-TB4-SC group) or IM (BCG/rAd-TB4-IM group) injections 10 weeks after BCG priming (Table 1). Lung lymphocytes in the SC and IM groups stimulated with the Ag85B peptide produced more IFN-γ than those in the BCG group. Similarly, IFN-γ production in splenocytes of the SC and IM groups significantly increased compared to that in those of the BCG group (Figure 3A).

**TABLE 1:** Immunization groups for the efficacy test vaccinated via various administration routes.

Groups (*n* = 5 in each)	Prime (day 0)/SC	1st boost (10 weeks)		2nd boost (14 weeks)	
	**Vaccine (2 × 10^5^ CFU/mouse)**	**Vaccine**	**Route, dose (IFU/mouse)**	**Vaccine**	**Route, dose (IFU/mouse)**
1	BCG	–		–	
2	BCG	rAd-TB4	SC, 1 × 10^7^	rAd-TB4	SC, 1 × 10^7^
3	BCG	rAd-TB4	IM, 1 × 10^7^	rAd-TB4	IM, 1 × 10^7^

SC, subcutaneous; IM, intramuscular; CFU, colony-forming unit; IFU, infectious virus unit; BCG, Bacillus Calmette–Guérin.

Based on the multifunctional features of rAd-TB4 (Figure 2), we quantified the frequencies of cytokine (IFN-γ, TNF-α, and IL-2) responses, which comprised three (3+), two (2+), or one (1+) cytokine(s) in both the lungs and spleen. In the case of lung lymphocytes, SC immunization of rAd-TB4 elicited a higher population of 3+ cytokine-secreting CD4^+^ and CD8^+^ T cells compared to IM immunization. IM immunization showed identical levels of BCG groups (Figure 3B). Similarly, in CD4^+^ T cells of splenocytes, the highest proportion of 3+ T cells was observed in SC-immunized mice. In the case of CD8^+^ T cells, however, it was difficult to compare the differences between the three groups (Figure 3C). Moreover, among cytokine-secreting T cells, the proportion of 3+ T cells was highest in the BCG/rAd-TB4-SC group. In contrast, BCG and rAd-TB4-IM groups elicited only marginal T-cell responses in both organs (Figures 3B,C, pie chart).

**FIGURE 4:**
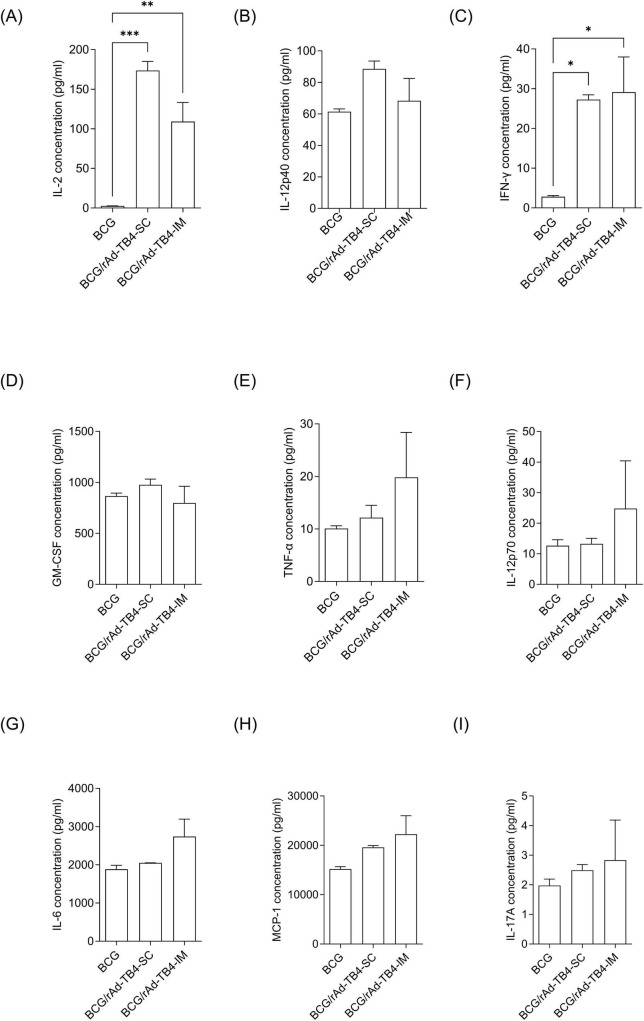
Comparison of inflammatory cytokine production in lung cells following immunization using different routes. Mice in each group were immunized via the SC or IM route. One week after the final immunization, the mice were euthanized, and lung lymphocytes were harvested. Cultured supernatants of lung lymphocytes were collected for cytokine level measurement after stimulation with the Ag85B peptide (100 ng/mL) for 36 h at 37°C. Production levels of nine cytokines **(A)** IL-2, **(B)** TNF-α, **(C)** IFN-γ, **(D)** GM-CSF, **(E)** IL-12p40, **(F)** IL-12p70, **(G)** IL-6, **(H)** MCP-1, and **(I)** IL-17A were examined using a bead-based multiplex cytokine assay. Statistical analysis was performed using the unpaired Student’s *t*-test; *p < 0.05, **p < 0.01, ***p < 0.001 compared with the BCG group.

Next, lung lymphocytes of rAd-TB4-vaccinated mice were stimulated *ex vivo* to profile Th1-, Th2-, and Th17-related cytokine productions (Figure 4). The production of Th1-related cytokines (IFN-γ, TNF-α, and IL-2) was elevated in both SC and IM groups compared to that in the BCG group. The increases in IFN-γ and IL-2 production were more significant in the SC group (*p* < 0.0001 for both) than those in the IM group (*p* < 0.05 for both), whereas the increase in TNF-α was not significant. The level of IL-17A, which has recently attracted attention for its essential role in vaccine-induced immune responses, also increased in the SC group compared to that in the BCG group; however, the increase was not significant. In addition, representative pro-inflammatory cytokines, such as granulocyte-macrophage colony-stimulating factor (p > 0.05) and IL-12p40 (p < 0.01) were induced after SC administration of rAd-TB4 (Figure 4).

### 3.3 Protective efficacy of rAd-TB4 in the *M. tuberculosis*-infected mouse model

**FIGURE 5:**
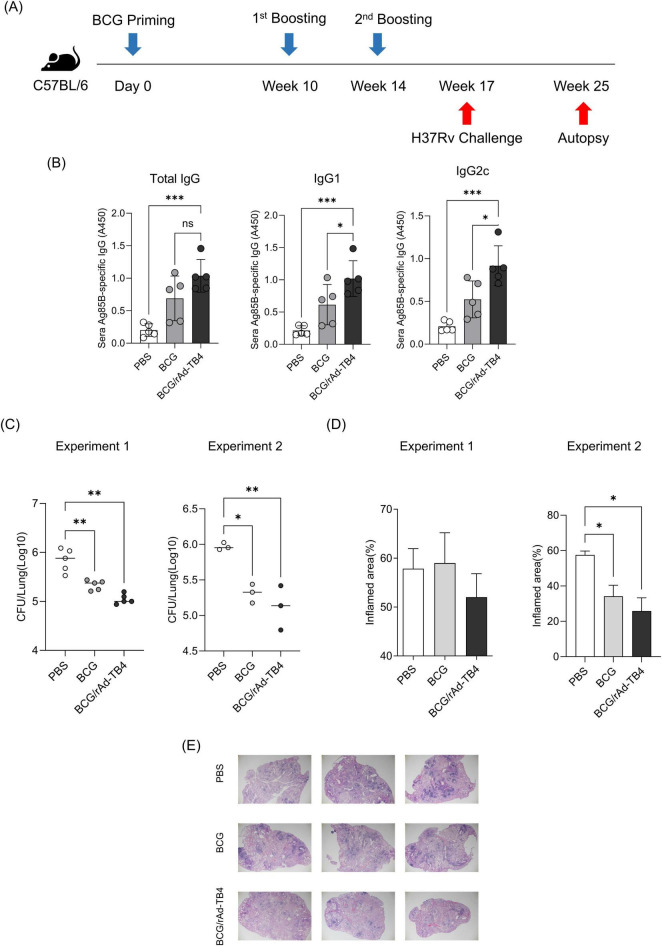
Protective efficacy of rAd-TB4 against H37Rv aerosol challenge. **(A)** Schematic of the immunization schedule and subsequent evaluation. The SC-vaccinated mice were challenged with H37Rv via the aerosol route (*n* = 5 or *n* = 3 per group). **(B)** After 8 weeks, the mice sera were collected and measured Ag85B specific total IgG, IgG1, and IgG2c. **(C)** Lung bacterial loads 8 weeks after H37RVchallenge. **(D)** The percentage of inflamed area of the lung. **(C,D)** Conducted twice. **(E)** Representative H&E staining images from the lungs of Experiment 1. Statistical analysis was performed using analysis of variance, followed by Dunnett’s multiple comparisons; *p < 0.05, **p < 0.01, ***p < 0.001. Data are means ± SEM.

**FIGURE 6:**
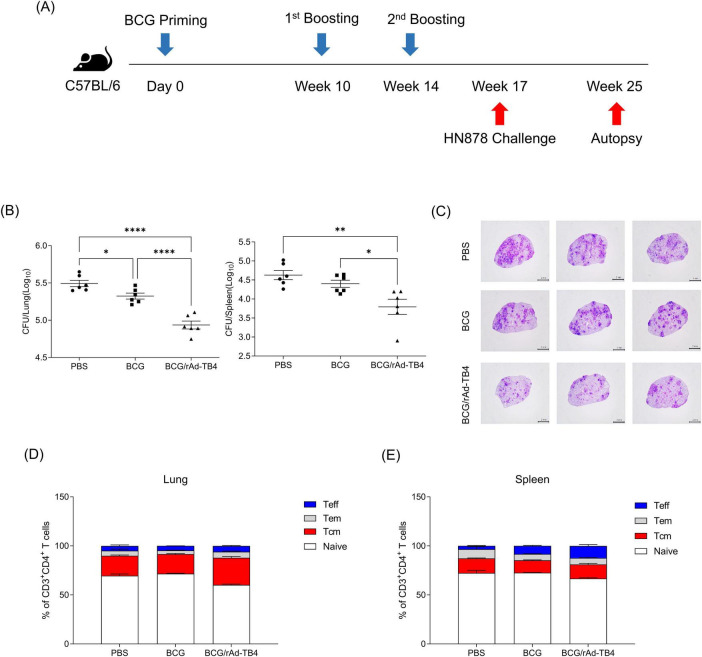
Protective efficacy of rAd-TB4 against clinical isolated strain HN878. **(A)** Schematic of the immunization schedule and subsequent evaluation. The SC-vaccinated mice were challenged with HN878 via the aerosol route (*n* = 6). **(B)** Lung and spleen bacterial loads 8 weeks after HN878 challenge. **(C)** Representative H&E staining images of infected lungs. **(D)** Lung lymphocytes and **(E)** splenocytes of infected mice were stimulated with Ag85B peptide for 5 h at 37°C. Frequency of Naïve T cell (CD44^–^ CD62^+^), Tcm (CD44^+^ CD62^–^), Teff (CD44^+^ CD62^–^ CD17^–^), Tem (CD44^+^ CD62^–^ CD17^+^) were presented along with the median. Data show the mean ± standard deviation from triplicate wells in each group; *p < 0.05, **p < 0.01, ****p < 0.0001 obtained using ordinary two-way ANOVA.

Next, we challenged C57BL/6 mice (*n* = 5) with the *M. tuberculosis* H37Rv strain via aerosol exposure to evaluate the protective efficacy of rAd-TB4. Eight weeks after infection, we collected mouse serum to measure antigen specific IgG titer and the mouse lungs to determine the bacterial load in each individual animal and stained them with H&E to measure the area occupied by inflammation (Figure 5A). Inflamed area and CFU measurements were conducted twice to verify protective effectiveness. Along with the above T-cell response and cytokine production result followed by SC immunization, BCG/rAd-TB4 immunization produced a significantly higher Ag85B-specific IgG and IgG1 compared with BCG immunization (Figure 5B). Notably, IgG2c production which is engaged in Th1 response was induced in rAd-TB4 immunization. In the case of Experiment 1, BCG/rAd-TB4 immunization elicited a significant protective effect against H37Rv (5.013 log_10_) compared to that with PBS (5.889 log_10_) or BCG (5.342 log_10_) immunization (Figure 5C). The histopathology result showed reduction in the inflamed area of BCG/rAd-TB4 mice groups; however, no significant differences were present between the unvaccinated and BCG-vaccinated groups (Figure 5D). Due to the inconsistent results observed in the inflamed area, additional experiments were conducted to revalidate these findings. C57BL/6 mice (*n* = 3) were challenged with the H37Rv strain via identical challenge method. In Experiment 2, the rAd-TB4 boosting resulted in a reduction of both the inflamed area and CFU values compared to those of the PBS and BCG groups (PBS: 5.970 log_10_; BCG: 5.314 log_10_; BCG/rAd-TB4: 5.118 log_10_) (Figures 5C,D). Representative images of H&E staining lung images from Experiment 1 also showed reduced inflammatory regions (Figure 5E). To evaluate the protective efficacy of rAd-TB4, a challenge study was conducted using the clinical isolated strain HN878. C57BL/6 mice (*n* = 6) were challenged with HN878 following an identical regimen and infection schedule of H37Rv challenge experiment (Figure 6A). Eight weeks post-infection, lungs and spleens were harvested to assess bacterial load and to identify T cell phenotype analysis. The BCG/rAd-TB4 group exhibited a significantly lower bacterial burden in the lungs and spleens compared to the BCG-only group (Figure 6B) and showed reduced inflammatory lesions in the rAd-TB4 boosted group (Figure 6C). To further characterize the cellular response to *M. tuberculosis* infection, a T cell phenotype analysis was performed on CD4^+^ T cells isolated from lung lymphocytes and splenocytes. To identify phenotypic differences, we analyzed the distributions of the canonical CD4^+^ T cell subsets. Lung lymphocytes of rAd-TB4 boosted mice contained significantly higher numbers of Ag85B-specific central memory T (Tcm) cell and effector memory T (Tem) cell compared to those in BCG vaccinated mice (Figure 6D). Notably, splenocytes from rAd-TB4 boosted mice demonstrated a remarkable increase in the effector T (Teff) cell population (Figure 6E).

### 3.4 Transcriptome profiling of rAd-TB4 responses in BCG-primed mice

**FIGURE 7:**
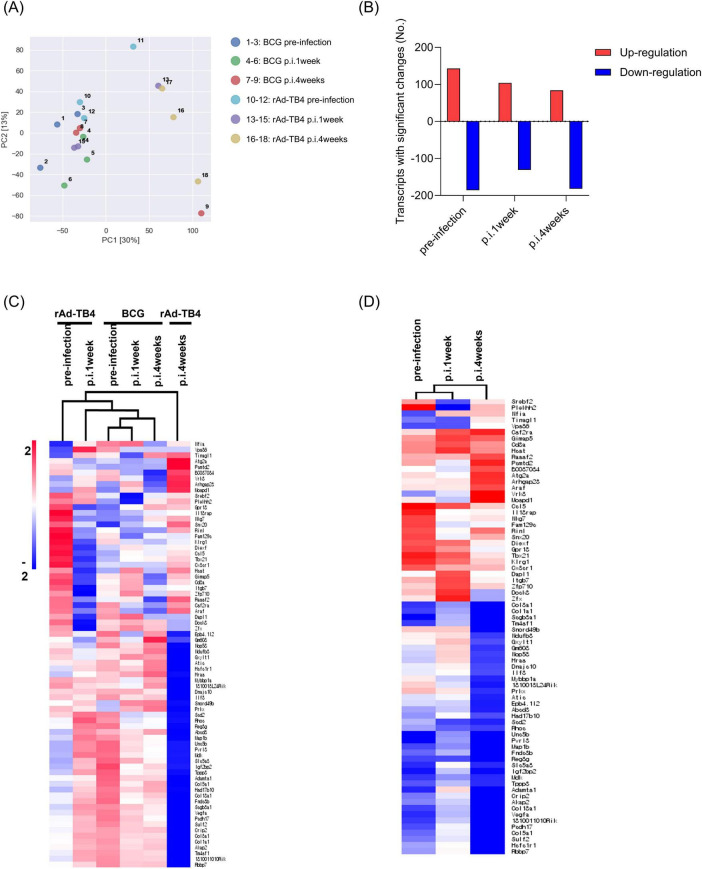
Transcriptional profiles and hierarchical clustering analysis of differentially expressed genes (DEGs). **(A)** Principal component analysis (PCA) of 18 samples using single normalized mRNA expression levels of three replicate samples in each group. **(B)** Bar graph showing significantly [fold change, 2; normalized data (log_2_), 2; and *p*-value, 0.05] up- or down-regulated genes between rAd-TB4 vs. BCG groups. Hierarchically clustered heat map calculated with Euclidean distance to confirm gene expression profiles in **(C)** respective groups and **(D)** rAd-TB4 group compared with BCG group at each time point: pre-infection, 1 week post-infection (p.i.1week), and 4 weeks post-infection (p.i.4weeks).

Next, we determined the transcriptome profile associated with BCG/rAd-TB4 immunization in *M. tuberculosis*-infected mice 12 weeks post-challenge. Whole blood samples collected at pre-infection, 1 (p.i.1week) and 4 weeks post-infection (p.i.4weeks) from both groups were used for RNA isolation and transcriptome profiling. PCA using RNA-sequencing (RNA-seq) data revealed clustering of all groups, except for the rAd-TB4 p.i.4weeks group (Figure 7A). We identified 23282 DEGs [fold change, 2.0; normalized data (log_2_), 2.0; p ≤ 0.05] using 18 RNA-seq libraries (triplicates for each group). Among these DEGs, 143, 104, and 84 genes were significantly upregulated, and 186, 131, and 182 genes were significantly downregulated in the rAd-TB4-immunized group at pre-infection, p.i.1week, and p.i.4weeks, respectively, compared to those of the respective BCG-administered groups (Figure 7B; Table 2). Normalized data from each group were hierarchically clustered to gain further insight. The BCG-administered groups at pre-infection and p.i.1week showed minimal changes, while gene expression was most variable in the rAd-TB4-immunized group at p.i.4weeks compared to those in pre-infection and p.i.1week (Figure 7C). In addition, we hierarchically clustered the data based on the fold-change between the rAd-TB4- and BCG-vaccinated groups at each time point. Gene expression dynamics were more discrete at p.i.4weeks than at the other two time points (Figure 7D). These results demonstrated that the expression pattern of the transcriptome changed over time after the *M. tuberculosis* challenge in the rAd-TB4-vaccinated group.

**TABLE 2:** Total number of up- and downregulated differentially expressed genes (DEGs) in rAd-TB4-vaccinated mice compared with those in BCG-vaccinated mice.

rAd-TB4 vs. BCG			Upregulated genes (n)	Downregulated genes (n)	Total DEGs (n)
C57BL/6	Pre-challenge		143	186	23282
	Post-challenge	1 week	104	131	
		4 weeks	84	182	

BCG, Bacillus Calmette–Guérin.

### 3.5 Classification of expressed genes engaged in immune responses

**FIGURE 8:**
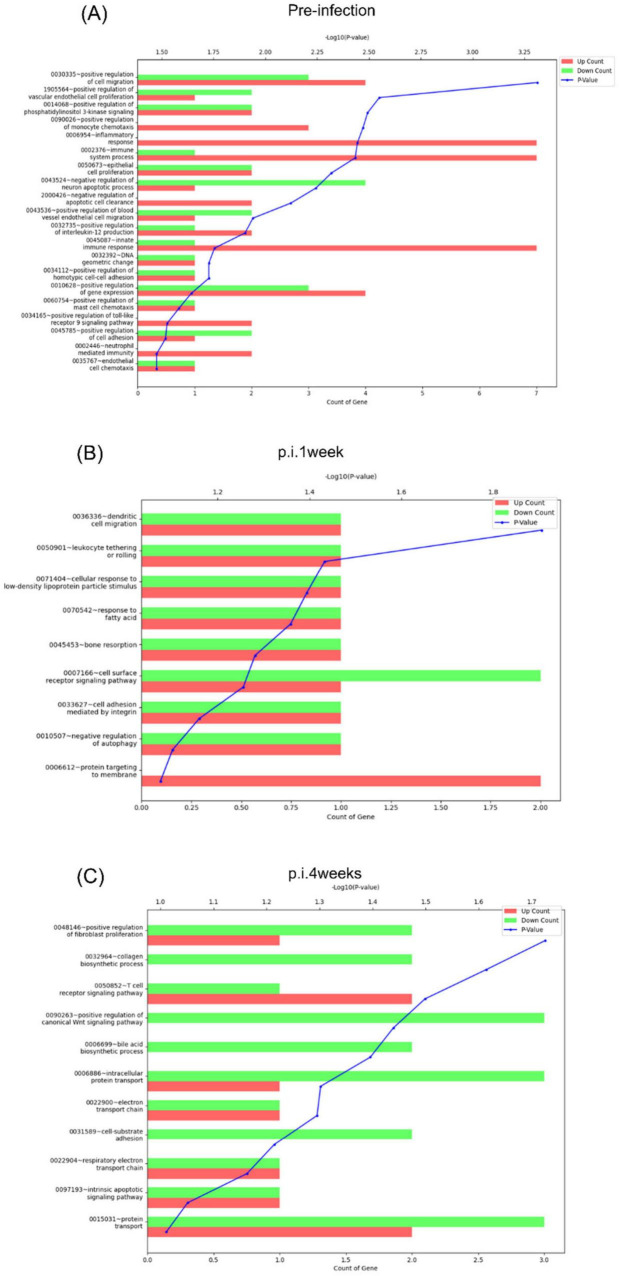
Functional annotation analysis of the DEGs at each time point. Functional annotation analysis of the DEGs [fold change: 2, normalized data (log_2_): 4, *p*-value: 0.05] identified between rAd-TB4 vs. BCG based on gene ontology (GO) was performed using the DAVID 6.7 database at **(A)** pre-infection, **(B)** 1 week post-infection (p.i.1week), and **(C)** 4 weeks post-infection (p.i.4weeks).

GO functional enrichment analysis of the DEGs identified in the BCG- and rAd-TB4-vaccinated mice was performed to screen for specific expression during infection. Among the top 10 enriched biological processes during pre-infection, “innate immune response,” “inflammatory response,” and “positive regulation of cell migration (Matsumiya et al., 2014; Santoro et al., 2018)” were positively regulated in rAd-TB4-vaccinated mice compared with those in the BCG-vaccinated mice (Figures 8A,B). At p.i.4weeks, “positive regulation of the canonical Wnt signaling pathway” was downregulated in the rAd-TB4-vaccinated groups compared to that in the BCG-vaccinated group (Figure 8C). We compared the mRNA expression levels of genes related to the canonical Wnt/β-catenin pathway in rAd-TB4-vaccinated mice and BCG-vaccinated mice to precisely identify the affected signaling moieties. The findings demonstrated that *vps35*, *col1a1*, and *sulf2* were downregulated in rAd-TB4-vaccinated mice compared to those in BCG-vaccinated mice at p.i.4weeks (Supplementary Figure S3A). Moreover, these genes showed reduced fold changes after the pathogen challenge (Supplementary Figure S3B). These observations indicate that rAd-TB4 immunization engages in down-regulation of canonical Wnt/β-catenin pathway.

## 4 Discussion

The WHO recommends a single dose of the BCG vaccine, citing insufficient evidence to support the effectiveness of additional doses and as reported in clinical study conducted in Malawi, BCG re-vaccination does not provide significant protection against overall tuberculosis (Glynn et al., 2021). Based on these findings, we did not include a repeated BCG vaccination regimen as a control group and evaluated rAd-TB4 effectiveness as a BCG-booster vaccine.

In this study, BCG-primed rAd-TB4 immunization showed good immunogenicity and protection against the *M. tuberculosis* challenge compared to those with BCG immunization alone. The rAd-TB4 candidate vaccine induced both CD4^+^ and CD8^+^ T-cell responses, which play essential roles in preventing pathogen invasion by inducing the production of pro-inflammatory cytokines, such as IFN-γ, TNF-α, and IL-2. This result indicated that a combination of multiple antigens expressed at different phases of infection can induce T-cell responses. Moreover, rAd-TB4 has been implicated in the induction of *M. tuberculosis*-specific IgG production, implying a B-cell response. Together, these results demonstrated the immunogenic capability of the recombinant adenovirus-based vaccine, rAd-TB4, as a BCG booster vaccine in a mouse model. Additionally, the present study provides evidence that the SC route enhances CD4^+^ and CD8^+^ T lymphocyte responses and suggests the optimal strategy for administrating rAd-TB4 vaccination.

The rAd-TB4 multi-antigenic candidate developed in this study included the whole sequences of ESAT-6, Ag85B, and Rv2660c and a partial sequence of MPT64 (residues 190–198). These four mycobacterial antigens and peptides included in the rAd-TB4 recombinant covering various stages of TB infection—Ag85B, ESAT-6, and MPT64—represent early secreted antigens, whereas Rv2660c is associated with late stages of infection (Brandt et al., 2000; Huygen, 2014; Yihao et al., 2015; Babaki et al., 2017; Cao et al., 2021). Ag85B is the major immunogen as it enables bacteria to avoid the host immune response by the interference of phagolysosome formation for host defense (Babaki et al., 2017), and ESAT-6 is an early secreted protein and virulence factor, which is used as a diagnostic tool (Brandt et al., 2000; Jung et al., 2017; Yu et al., 2023). Additionally, MPT64 is one of the secretory proteins of *M. tuberculosis* and highly detected in the serum and sputum of active patients with TB (Mehaffy et al., 2017). Although it is widely used for vaccine candidate and diagnosis tool, it could act as immunosuppressor which inhibit the apoptosis and modulates DC differentiation to myeloid-derived suppressor cells to inhibit pro-inflammatory response (Wang et al., 2014; Singh et al., 2022). In this study, we designed vaccine candidate with partial epitope region of MPT64 covered 190–198, which has an immunogenic characteristic. The peptide of residues 190–198 of MPT64 promotes CD8^+^ T-cell responses through presentation by antigen-presenting cells after BCG vaccination or *M. tuberculosis* infection (Feng et al., 2001; Mustafa and Shaban, 2010) and we expect that the partial epitope region of MPT64 will trigger immune response starting from the T cell receptor that other potentially protective antigens than the four tested have been reported previously (Ivanyi and Ottenhoff, 2014) and emphasized the importance of containing MHC-HLA-permissive epitopes (Ivanyi, 2014), which needs to be examined with the reported Ad5 vaccine.

To circumvent the complex pathogenesis of *M. tuberculosis*, multiphasic and multi-antigen vaccine candidates have been developed that showed their effectiveness in animal models and clinical trials, including H56 (comprising Ag85B, ESAT-6, and Rv2660c) (Aagaard et al., 2011; Jenum et al., 2021) and ID93 (comprising Rv2608, Rv3619, Rv3620, and Rv1813) (Day et al., 2021). MVA85A is used as a BCG booster prophylactic vaccine with promising immunogenicity in animal models; however, it showed inadequate protective evidence in clinical trials (Tameris et al., 2013). Although the exact mechanism underlying the protective effectiveness of MVA85A is not known, its clinical failure is speculated to be because of its low antigen complexity, which consequently led to the discovery of various vaccine candidates with antigenic diversity.

We established the optimal vaccination route and dosage for rAd-TB4 using a prime-boost vaccination strategy that demonstrated the highest efficacy (Supplementary Figures S4, S5). We immunized mice with the rAd-TB4 via four routes: SC, IM, intranasal (IN), and intraperitoneal (IP) and different dosages ranged between 1 × 10^6^ to 1 × 10^8^, in the absence of BCG (Supplementary Figures S4, S5). In the lung lymphocytes, IN route prompted an immunogenic response, whereas immunization via the SC route prompted a response in splenocytes (Supplementary Figure S4). As a result, we selected the SC and IM routes for the immunogenicity analysis to induce systemic immune response. However, considering recent studies on the mucosal immunity induction potential of IN administration of adenovirus-vectored vaccines (Xing et al., 2009; Afkhami et al., 2023), further research on IN vaccinated rAd-TB4 could be considered as a promising TB vaccine.

Nevertheless, mice immunized via the SC route produced “protective” cytokines and were effectively protected against the H37Rv aerosol challenge. Building on these observations it was confirmed that SC vaccination with rAd-TB4 as a BCG-booster results in a reduction of CFU compared to BCG vaccination alone, though no significant difference in the inflamed area, indicative of lymphocyte aggregation, was observed between the unvaccinated (PBS group) and BCG vaccinated groups in Experiment 1. However, the pulmonary bacterial load, a direct indicator of the immune defense capability, showed a reduced CFU count in the lungs of rAd-TB4-boosted mice, thereby confirming the potential of rAd-TB4 as an effective BCG booster vaccine. The C57BL/6 model, while progressing with inflammatory cell infiltration during TB infection, does not develop caseous necrosis, thus differing morphologically from the granuloma seen in human patients with active TB.

Additionally, the absence of caseous necrosis prevents inter-mouse transmission of tuberculosis, highlighting a limitation that necessitates the development of an improved infection model (Rhoades et al., 1997; Cosma et al., 2003; Ramakrishnan, 2012; Silva Miranda et al., 2012; Hunter et al., 2023). This study measured only the extent of lymphocyte aggregation, but future research should refine the measurement of specific infiltrate of inflammatory cells, such as peribronchiolitis and alveolitis, to more accurately assess the onset of pulmonary inflammation (Kwon et al., 2022), or utilize mouse strains that exhibit lesions similar to caseous necrosis for enhanced analysis of defensive capabilities (Huynh et al., 2011; Hunter et al., 2023).

Among various vaccine platforms, virus-vectored vaccines are expected to induce robust and long-lasting immune responses (Afkhami et al., 2016; Sayedahmed et al., 2018; Afkhami et al., 2023). In TB vaccine development, several candidates for preclinical and clinical trials have been constructed using viral vectors, such as modified vaccinia virus or adenovirus (Tameris et al., 2015; Feng et al., 2020; Koch et al., 2020; Vierboom et al., 2020). Human adenovirus-vectored vaccines have been adopted for several diseases and to elicit CD8^+^ and CD4^+^ T-cell responses, which are only marginally induced by BCG immunization (Wang et al., 2004; Xing et al., 2009; Sayedahmed et al., 2018; Zhang et al., 2018). However, pre-existing immunity to adenoviruses in some individuals may reduce vaccine efficacy (McCoy et al., 2007; Sharma et al., 2010). Recently, an Ad5-vectored coronavirus disease (COVID-19) vaccine developed by CanSino Biologics (China) has been evaluated in a phase II trial in healthy adults ≥ 18 years, followed by final efficacy and interim safety evaluation in a phase III trial. The vaccine demonstrated good-tolerance and immunogenicity, indicating the potential of Ad5-vectors for constructing recombinant vaccines. Furthermore, an additional dose with a flexible term, heterologous prime-boost immunization, and a high dosage potentially overcame the pre-existing immunity against Ad5 (Zhu et al., 2020; Halperin et al., 2022). Therefore, we speculate that heterologous immunization using rAd-TB4 or other candidates constructed with other antigens or viral vectors for TB prevention could be effective in humans.

We also analyzed the transcriptome of rAd-TB4- and BCG-vaccinated mice to reveal the gene expression profiles at different time points following the *M. tuberculosis* challenge. As evidenced by the PCA and hierarchical clustering analysis, the genes in rAd-TB4-vaccinated mice at p.i.4weeks showed a distinct expression pattern compared to that in rAd-TB4 and BCG-vaccinate mice. GO analysis of DEGs in rAd-TB4-vaccinated mice revealed similar results of functional classification as obtained in human patients with TB, including DEGs associated with “nucleotide binding,” “signaling molecules,” and “protein transport” (Alam et al., 2019). Wnt signaling mediates immunomodulatory functions during inflammation and pathogen infection and induce pro- or anti-inflammatory responses depending on the cellular and cytokine environments (Kuncewitch et al., 2013; Wadey et al., 2022). Although there are several unestablished roles in infectious environments, the activation of β-catenin-dependent Wnt signaling limits pro-inflammatory responses during *M. tuberculosis* infection (Brandenburg and Reiling, 2016). Several studies have reported that Wnt3A and Wnt6 skew the intracellular milieu of infected macrophages toward an anti-inflammatory cytokine profile during *M. tuberculosis* infection (Schaale et al., 2013; Villaseñor et al., 2017; Jati et al., 2019). Moreover, inhibition of Wnt/β-catenin signaling is implicated in the reduction of RNA virus replication such as SARS-Cov-2 (Xu et al., 2024). In this study, we showed that rAd-TB4 immunization downregulated the positive regulation of the canonical Wnt signaling pathway and mRNA expression levels of the Wnt signaling pathway regulator at p.i.4weeks compared to those with the BCG vaccination. Based on these results, we suggest that the Wnt signaling pathway or its related pathway may be associated with protective effectiveness of rAd-TB4 via alteration of cellular niche toward pro-inflammatory cytokine signature. The exact role of this suggestion needs to be validated in our future studies such as comparison between the transcriptomic profile and CFU counts to profile immune correlates of protection. In addition, testing of mice for T-cell immunity was done only 1 week after rAd-TB4 vaccine boosting and protection against H37Rv infection only 3 weeks after rAd-TB4 vaccine boosting. This is a long way from the conditions in most humans, where active lung disease of adults develops several years decades after infection in young age.

Additionally, mice were tested only for protection against primary aerosol H37Rv infection. However, most *M. tuberculosis* infected humans develop latency/dormancy after primary infection and only a minority develop active pulmonary disease by re-activation of their latent infection. Hence, it is essential to test candidate TB vaccines in the established mouse models for protection against re-activation of latent infection (Cox et al., 1989). And also, the rAd-TB4 vaccine was tested only in BCG-primed mice. However, some “decoy” constituent within BCG may compromise against protection (Ivanyi, 2021). Therefore, it would be desirable to evaluate, if the rAd-TB4 vaccine would protect mice without BCG priming.

Furthermore, this study requires further improvement. A study comparing protection at 4 weeks and 20 weeks post *M. tuberculosis* infection has shown that while the efficacy of BCG declines by 20 weeks, the protective capacity of the vaccine candidates remains intact (Nisa et al., 2024). Based on these results, it is essential to evaluate the long-term protective efficacy of rAd-TB4.

In summary, we demonstrated that rAd-TB4 induced an immune response following stimulation with TB-specific antigens. Vaccine administration via the SC route produced the highest immunogenicity, including CD4^+^ T-cell responses concomitantly producing IFN-γ, TNF-α, and IL-2. Our study showed that although the CFU values decreased in response to the H37Rv challenge, there was no significant reduction in the inflamed area. This result implies no sufficient evidence for the protective effectiveness of rAd-TB4. To address this limitation, we conducted an analysis using the clinical isolated strain HN878 to verify CFU values and further performed T cell phenotype analysis to assess the immune response related to protective efficacy, confirming the protective effectiveness. These results showed that rAd-TB4 is effective against adult pulmonary TB in a BCG-primed mouse model, rendering rAd-TB4 as a novel potential TB vaccine candidate against adult pulmonary TB. However, owing to the discrepancy in the immune response and mechanism of protection against TB between mice and humans, the efficacy of rAd-TB4 should evaluated in a guinea pig model that recapitulates features of disease progression (Clark et al., 2014) as in humans to precisely predict the protective effectiveness of rAd-TB4 in humans.

## Data Availability

The datasets presented in this study can be found in online repositories. The names of the repository/repositories and accession number(s) can be found at: https://www.ncbi.nlm.nih.gov/geo/query/acc.cgi?acc=GSE264141, GSE264141.

## Footnotes

1
https://mycobrowser.epfl.ch/

2
http://david.abcc.ncifcrf.gov/
