# Erratum: Immunogenicity and protective efficacy of a multi-antigenic adenovirus-based vaccine candidate against *Mycobacterium tuberculosis*

**DOI:** 10.3389/fmicb.2025.1605861

**Published:** 2025-05-22

**Authors:** Frontiers Production Office

**Affiliations:** Frontiers Media SA, Lausanne, Switzerland

**Keywords:** pulmonary tuberculosis, BCG vaccine, adenovirus vector, multi-antigenic vaccine, immunization, mouse model, immune response

Due to a production error, Figure 8 was not included in the article. Figure 8 and its caption appears below.

**Figure 8:**
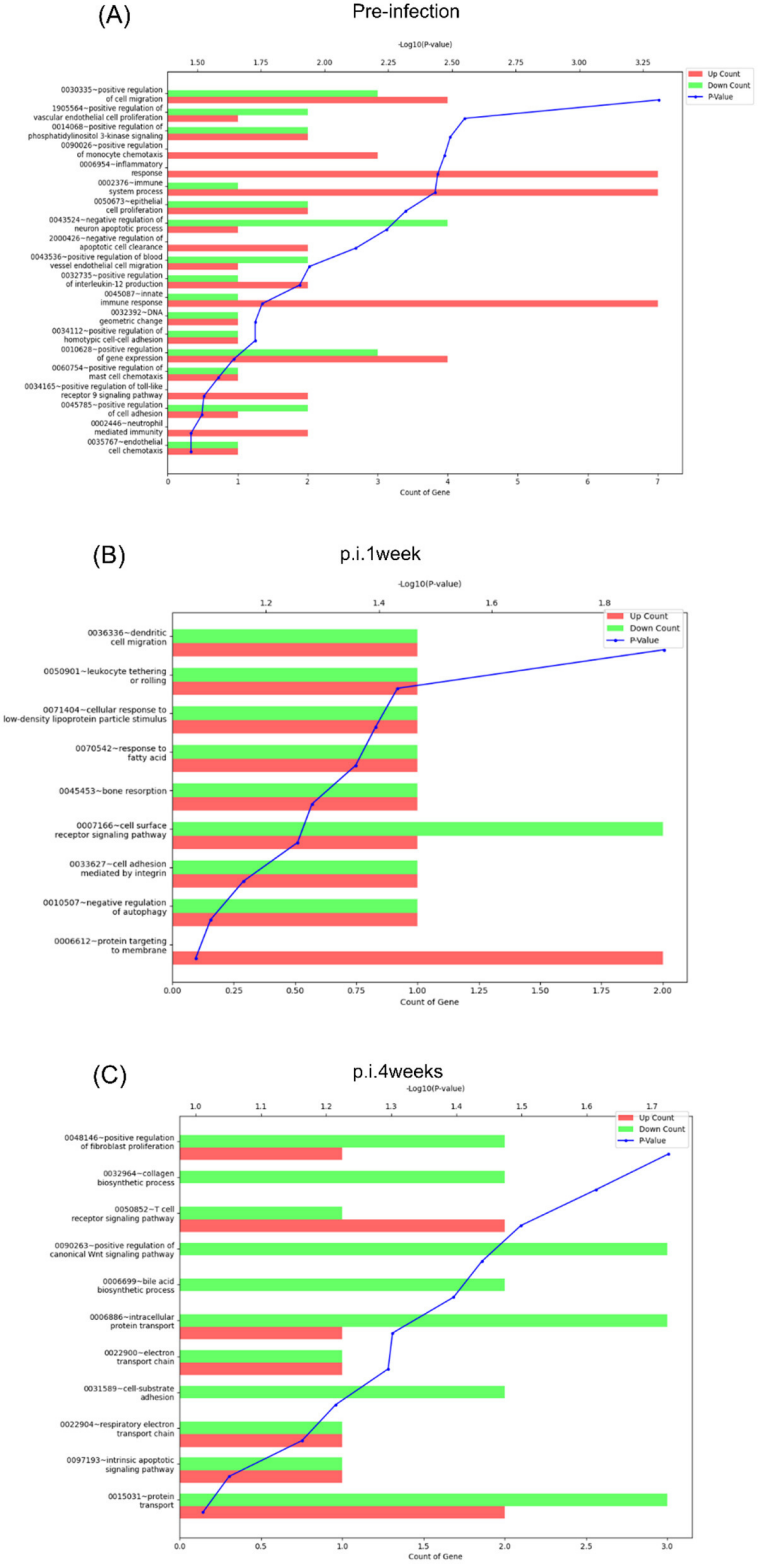
Functional annotation analysis of the DEGs at each time point. Functional annotation analysis of the DEGs [fold change: 2, normalized data (log_2_): 4, *p*-value: 0.05] identified between rAd-TB4 vs. BCG based on gene ontology (GO) was performed using the DAVID 6.7 database at **(A)** pre-infection, **(B)** 1 week post-infection (p.i.1week), and **(C)** 4 weeks post-infection (p.i.4weeks).

The publisher apologizes for this mistake. The original version of this article has been updated.

