# Supplementary material for: Immunogenicity and protective efficacy of a multi-antigenic adenovirus-based vaccine candidate against Mycobacterium tuberculosis
Source: Front Microbiol. 2025 Jan 24;16:1492268. doi: 10.3389/fmicb.2025.1492268 (PMC11802578; doi:10.3389/fmicb.2025.1492268)
Supplement: Supplementary file 1 [file Data_Sheet_1.docx]

Supplementary Material

# Supplementary Figures


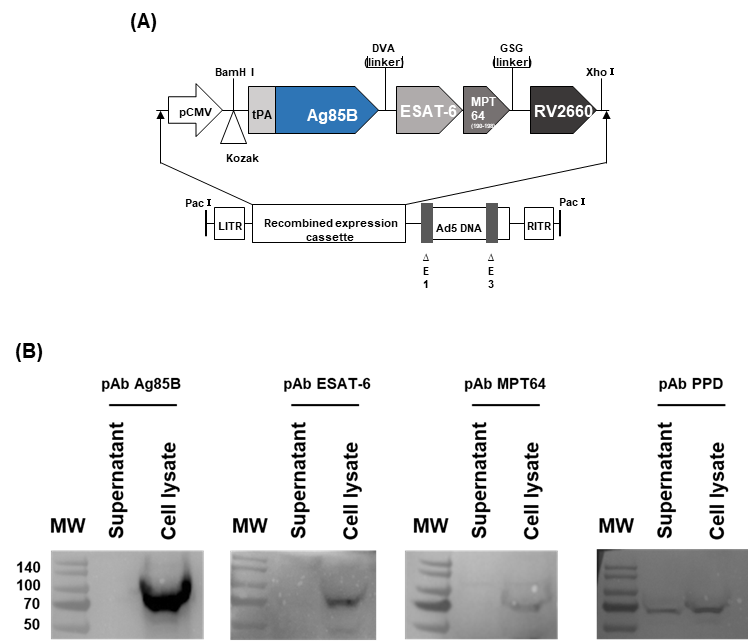


**Supplementary Figure 1. Molecular construction and in vitro antigen detection of rAd-TB4.** (**A**) The designing of multi-antigenic recombinant Ad5 strain (rAd-TB4). Four antigens Ag85B, ESAT-6, MPT64, and Rv2660 of Mycobacterium tuberculosis were inserted into the pAd/CMV/V5-DEST vector in the order Ag85B > ESAT-6 > MPT64 > Rv2660 and linked using a DVA and GSG linker. (**B**) Protein expression of rAd-TB4 in the infected HEK293A cell supernatants and cell lysate was examined using western blotting with polyclonal anti-Ag85B, ESAT-6, MPT64 and PPD antibody.


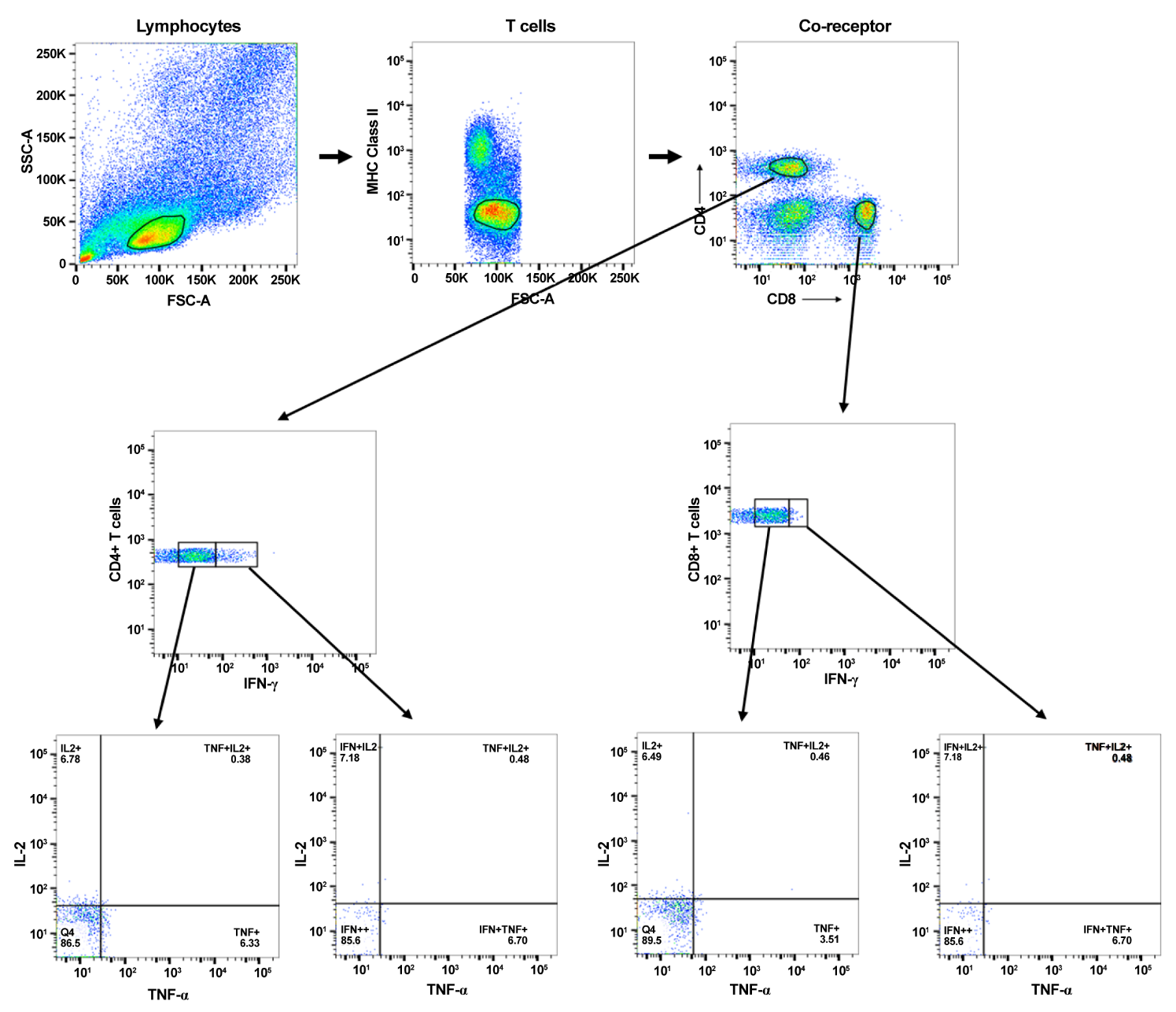


**Supplementary Figure 2. Gating strategy for the intracellular cytokine staining assay.** Lymphocytes were identified using an FSC-A vs. SSC-A plot, and T lymphocytes were selected based on negative major histocompatibility complex class II staining. Subsequently, CD4^+^ and CD8^+^ T-cells were identified using a CD4 versus CD8 dot plot followed by IFN-γ^-^ and IFN-γ^+^. FSC-A, Forward Scatter-A; SSC-A, Side Scatter-A; IFN-γ, Interferon-γ.





**Supplementary Figure 3. Expression patterns of genes associated with the Wnt signaling pathway.** Normalized amounts of Wnt signaling pathway-related genes were estimated for each group at p.i.4weeks. Target gene expression was normalized to that of the unvaccinated control group and adjusted for RNA yield (**A**) and fold changes in specific genes (**B**) related to the canonical Wnt pathway in rAd-TB4-vaccinated mice relative to BCG-vaccinated mice at each time point. The data represent normalized target gene levels. *Vps35, Vacuolar protein sorting ortholog 35; col1a1, Collagen type 1 alpha 1; sulf2, sulfatase 2.*


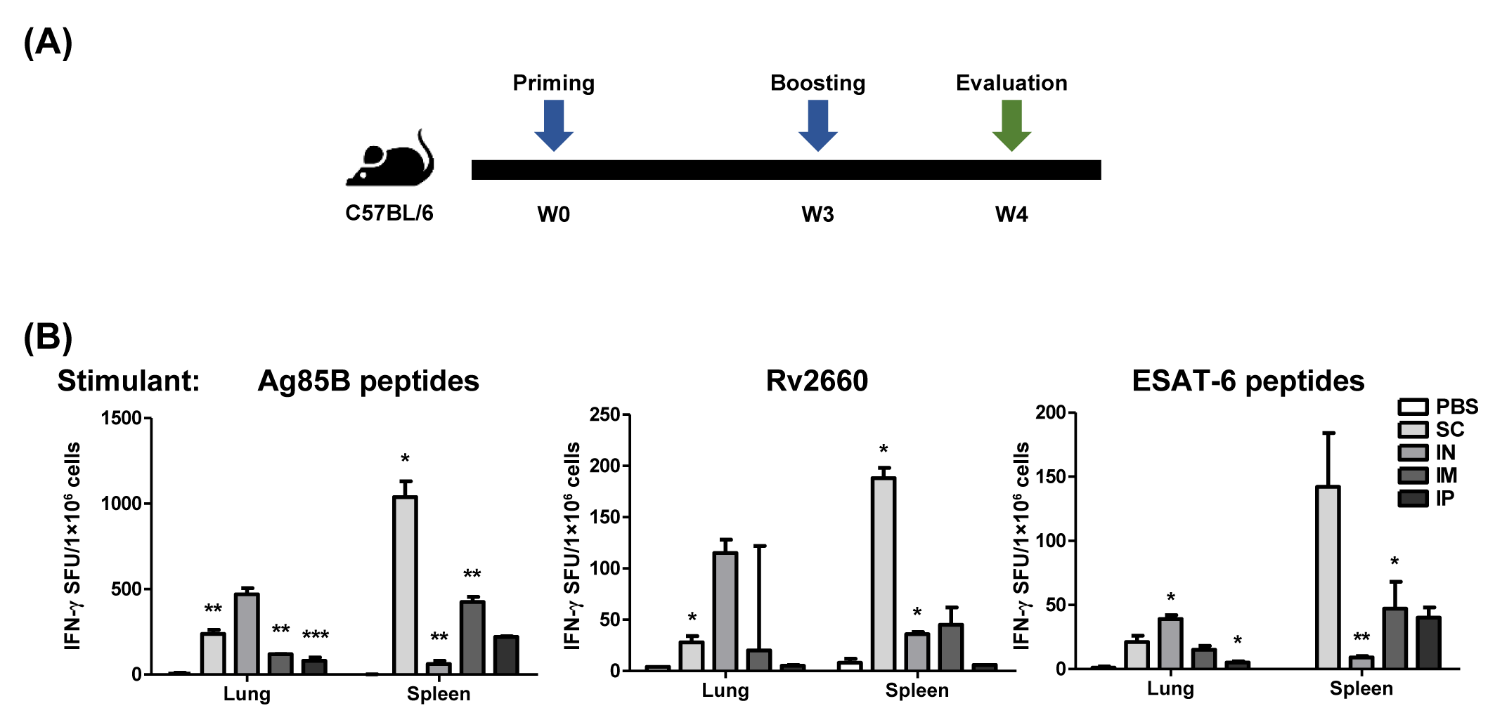


**Supplementary Figure 4. Immunogenicity of rAd-TB4 in the absence of BCG immunization.** (**A**) Schematic of the study design to evaluate immunogenicity in the absence of BCG, mice were vaccinated with rAd-TB4 twice at 3-week intervals (weeks 0 and 3). (**B**) After 1 week of boosting, the mice were necropsied to perform an ELISpot assay to measure IFN-γ secretion from lung lymphocytes and splenocytes following antigen stimulation. PBS, phosphate-buffered saline; SC, subcutaneous; IN, intranasal; IM, intramuscular; IP, intraperitoneal, ELISpot: Enzyme-Linked ImmunoSpot.


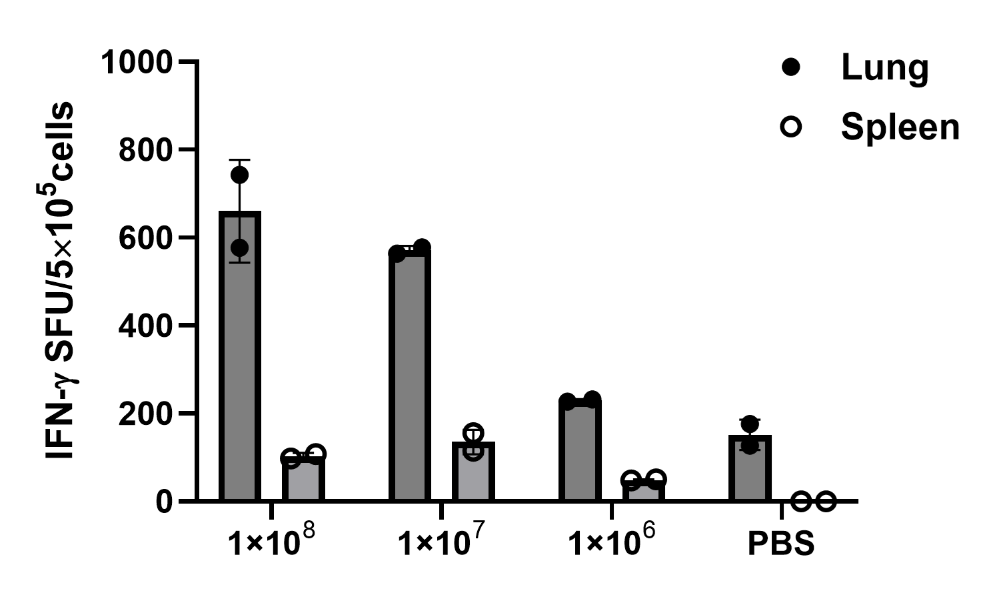


**Supplementary Figure 5. Immunogenicity for optimal immunization dose.** Mice were vaccinated with rAd-TB4 twice at 3-week intervals (weeks 0 and 3) with respective immunization dose (1×10^6^-10^8^ IFU/mice). The mice were autopsied to assess an ELISpot assay to measure IFN-γ secretion from lung lymphocytes and splenocytes following Ag85B stimulation after 1 week of last immunization.
